# DEPRESSION AMONG OLDER CANADIAN REFUGEES: THE PROTECTIVE ROLE OF SOCIAL SUPPORT

**DOI:** 10.1093/geroni/igz038.1980

**Published:** 2019-11-08

**Authors:** Esme Fuller-Thomson, Shen (Lamson) Lin, Karen Kobayashi, Simran R Arora, Hongmei Tong, Karen Davison

**Affiliations:** 1 University of Toronto, TORONTO, Ontario, Canada; 2 University of Victoria, Victoria, British Columbia, Canada; 3 MacEwan University, Edmonton, Alberta, Canada; 4 Kwantlen Polytechnic University, Surrey, British Columbia, Canada

## Abstract

This study’s objective was to identify which factors attenuate refugees’ higher odds of depression. A secondary analysis of 272 refugees and 29,398 non-refugees in the Canadian Longitudinal Study on Aging, a 2012 study of Canadians aged 45 to 85, was conducted. The prevalence of depression was higher among refugees than non-refugees (22.1% vs 15.2%, p<.001). The age-sex adjusted odds of depression for refugees (OR=1.70, p<.001) was only modestly attenuated when sociodemographic characteristics, physical health conditions, chronic pain, binge drinking and level of physical activity were taken into account (ORs ranged from 1.61 to 1.70, all p<.05). However, in the model adjusting for social support, the odds of depression for refugees was reduced to non-significance (OR=1.30, p=0.92). Refugees have higher odds of depression than non-refugees, and this excess vulnerability is associated with lower levels of social support. Targeted interventions to decrease isolation and improve refugees’ social support warrant greater attention

